# Data Diffraction: Challenging Data Integration in Mixed Methods Research

**DOI:** 10.1177/1558689816674650

**Published:** 2016-10-01

**Authors:** Emma Uprichard, Leila Dawney

**Affiliations:** 1 University of Warwick, Coventry, UK; 2 University of Brighton, Brighton, UK

**Keywords:** cut, mess, mixed methods, diffraction, integration

## Abstract

This article extends the debates relating to integration in mixed methods research. We challenge the a priori assumptions on which integration is assumed to be possible in the first place. More specifically, following Haraway and Barad, we argue that methods produce “cuts” which may or may not cohere and that “diffraction,” as an expanded approach to integration, has much to offer mixed methods research. Diffraction pays attention to the ways in which data produced through different methods can both splinter and interrupt the object of study. As such, it provides an explicit way of empirically capturing the mess and complexity intrinsic to the ontology of the social entity being studied.

The merits of mixed methods^1^ are now well recognized (Brannen, 1992; Bryman, 1984, 2006, 2007; Caracelli & Greene, 1993; Creswell, 2003; Fielding, 2012; Greene, 2007; Greene, Caracelli, & Graham, 1989; Greenhalgh et al., 2010; Huberman & Miles, 1983; Johnson & Onwuegbuzie, 2004; Johnson, Onwuegbuzie, & Turner, 2007; Pluye, Grad, Levine, & Nicolau, 2009; Tashakkori & Creswell, 2007; Tashakkori & Teddlie, 1998; Teddlie & Yu, 2007). This field has developed rapidly and there is now a proliferation of work that combines quantitative and qualitative methods to explore social phenomena. There is also a general consensus across that body of research that, whichever methods are used, integrating the mixed data is the desirable outcome. Although data integration is a sensible *goal*, we challenge the presupposition that it is necessarily the optimal *outcome* of mixed methods research. We do this by presenting an alternative to data integration based on the concept of *diffraction*, defined as a process of paying attention to the ways in which process produces “cuts” that can interrupt and splinter the object of study.

This challenge is made in eight methodical steps. Step 1 introduces integration in mixed methods work, highlighting the many different approaches and arguing that, while integration is a desired goal, it is not always successfully achieved in practice. Second, we suggest that authors typically respond to the difficulties of integration by reexamining the impact of different epistemologies relating to different methods, or by proposing that the research is conducted differently. In doing so, we question the assumption that integration is necessarily possible and suggest that, sometimes, integration may be problematic because of the object of research itself, which is complex, ontologically unstable, and may not be clearly bounded. Third, we outline a fundamental paradox at the heart of mixed methods research, namely, that mixed methods are assumed to be useful because of the complexity of the social world, yet in spite of this, it is also assumed to be both possible and desirable to integrate data relating to the study of complex, messy social objects. Steps 4 and 5 together introduce the notion of research as “cuts” and argue for an approach that acknowledges that data can “mess up” the object. Step 6 elaborates on this by drawing on Donna Haraway’s and Karen Barad’s work. We show that mixed methods have the capacity to produce sets of messy empirical “cuts” of the object being studied that do not always “cohere” (Barad, 2007) and that these cuts can be addressed through data “diffraction” (Haraway & Randolph, 1997). Step 7 reiterates that diffraction and integration can work together, but that data diffraction offers mixed methods researchers a means of empirically capturing some of the messiness of social objects, letting messy objects be messy in a way that data integration cannot. Finally, we reflect on the rich opportunities diffraction offers to mixed methods work in contemporary contexts where data are increasingly mixed.

To be clear, in arguing for diffraction as an alternative to integration, we not wish to negate or undo the efforts colleagues have made regarding data integration. Rather, we emphasize the need for an approach that explicitly supports instances where data do *not* integrate or “cohere” and argue that this may be due to the messy nature of the object of study. In doing so, we provoke a discussion around the orthodoxy of integration as a goal of mixed methods research.

## Data Integration in Theory and Practice

There has been a growing debate about how best to integrate quantitative and qualitative data, but the basic idea behind it is that, within a single study, the resulting analysis is done *across* or *through* the different data. Instead of “adding up” data, where findings of one method are considered alongside findings of another, integrating data goes beyond any individual method and considers the interaction—or synthesis—of data. As Fielding (2012) suggests, mixed methods allow for greater “analytic density.” The goal of data integration, then, can be seen to produce a (more) comprehensible object. Overall, the possibility of data integration lies in the extent to which data from different methods can be interpreted *together* in a meaningful way. In effect, data integration is seen as fundamental to what we do as mixed methods researchers. Fielding (2012) explains:

Integration is really the heart of the whole mixed methods exercise because the purpose of mixing methods is to get information from multiple sources and so the issues in bringing together the information are crucial (p. 127).

Given its prime place of importance in this area of methodological research, it is surprising, therefore, that integration has not received more attention. As Bazeley and Kemp (2012) note, “not only is integration of methods undertheorized and understudied but also the level of integration practiced in many mixed methods studies remains underdeveloped” (p. 56).

Where authors do focus on integration, they often underscore the sheer *challenge* of integrating different methods (Bryman, 2006, 2007; Caracelli & Greene, 1993; Fielding, 2012; Harrits, 2011; Mertens & Hesse-Biber, 2012; O’Cathain, Murphy, & Nicholl, 2007; Onwuegbuzie & Teddlie, 2003; Plano Clark, Huddleston-Casas, Churchill, O’Neil Green, & Garrett, 2008; Sandelowski, 2000; Teddlie & Tashakkori, 2008, 2012; Yin, 2006). Bryman (2007), for instance, shows that most studies using mixed methods write up the findings within each method separately and then attempt to integrate them in an analysis that produces a coherent narrative. The result, Bryman (2008) suggests, is that integration is often “not achieved and difficult to do” (p. 93).

Likewise, Bazeley and Kemp (2012) argue:

Typically, quantitative results, usually from surveys, are presented first in reports of studies, to be followed by a necessarily brief thematic analysis of interview material or answers to open-ended questions. Sometimes the threads from both strands are drawn together as a basis for a model or some other conclusion but not always. (p. 56)

There is a discrepancy, therefore, in terms of what the literature on combining methods encourages researchers to do *in theory* and what actually happens *in practice.*

We are not alone in noticing this discrepancy. Many respond to the difficulty of integrating findings by focusing on the epistemological issues of bringing together quantitative and qualitative approaches (Bryman, 1988, 2006; Caracelli & Greene, 1993), echoing the enduring philosophical and historical tensions that mixed methods research tends to bring. Some authors propose alternative philosophical approaches such as pragmatism or critical realism, as a way of resolving some of these mixed methods tensions. Yet explaining the difficulty of data integration through epistemological differences intrinsic to mixed methods does not resolve the problem: findings can be difficult to integrate within the *same* method too. Yet when that happens, the protocol within both quantitative and qualitative methods tends to be to *use* and *further explore* those contradictions rather than write them off as the product of incommensurate world views.

Rather than debating epistemological issues intrinsic to mixed methods research, some authors focus on the *practicalities* involved in the process of data integration itself. Here, findings that seem to offer very different representations of the object of research are positioned as problems that can ultimately overcome through more effective strategies of integrating data. Elliott (2005), for example, underscores the importance of *narrative* to provide a “reflexive bridge between the traditions of quantitative and qualitative methods” (p. 187). Creswell and Plano Clark (2007) propose that data are “brought together” through “merging”, “connecting” or “embedding” different data. Likewise, Creswell (2003) sketches the different configurations of actually *doing* mixed methods research and considers *when* and *where* in the research process data integration actually takes place. Others suggest various metaphors to help think through the process of data integration and what specific advantage it offers (see Bazeley & Kemp, 2012; Fielding, 2012). Fetters, Curry, and Creswell (2013) explicate this further by suggesting that integration is “implemented at the design, methods, and interpretation and reporting levels of research” (p. 2135). They also make the point that the “fit” of data integration can vary: “confirmation occurs when the findings from both types of data confirm the results of the other”; “expansion occurs when the findings diverge and expand insights”; “discordance” occurs when “findings are inconsistent, incongruous, contradict, conflict, or disagree with each other” (pp. 2143-2144). The list goes on; there are many more authors and approaches we might have cited.

## Challenging Integration

What brings these approaches together, though, is the a priori assumption that integration is both theoretically and practically feasible, so long as the epistemological differences can be overcome or the “right kind of approach” is used, and so on. The fact that integration is both always possible and desirable has been an enduring premise on which most mixed methods research has developed.

Our argument has less to do with how or where or when to integrate, although these may be contingent necessary conditions that make integration possible at all. Rather, we interrogate the very premise of integration in the first place. That is to say, an implicit assumption to integration is that the empirical data will depict a particular social phenomenon. Yet there is no a priori reason that this is necessarily so. Mixed data could equally reveal multiple phenomena that are entangled together, even though they appear to singular or whole. Mixed data could instead multiply the partiality, increase the uncertainty, and further entangle the subject. Our view is that mixed methods may confuse, split, fracture, trouble, or disturb what is (thought to be) studied and we need an alternative way of acknowledging this possibility. After all, we tend to assume that one method depicts one part or aspect of the object of study and if another method presents a different part or aspect, then the methods have together shown different parts or aspects of the same thing. But what if one method captures the “ear of the elephant” and another method captures the “tail of a mouse”? What if mixed methods, very successfully, capture *multiple* aspects of *multiple* parts that are entangled together instead of revealing some (singular) “thing” as “more” whole? There is no sure way of knowing whether empirical data are reflecting one or more objects; these are interpretations that are made *after the fact*.

What is missing for us within the mixed methods research is the possibility that it is sometimes problematic, and maybe even impossible, to integrate at all, not because data are generated from different methods, nor because of the different epistemological assumptions underpinning the methods, but importantly because of nature of the object of study itself. *Contradictory findings may be due to the ontological complexity of the object of study as much as the epistemological or methodological issues intrinsic to the overall research design. Furthermore, if we assume that social phenomena always exist in entanglements with other social phenomena, then we also need to assume the possibility that empirical work captures and may further complicate those entanglements.* A diffractive approach to data integration not only highlights *difference* but also highlights the “entangled nature of differences that matter” (Barad, 2007, p. 36).

## Mixed Methods’ Paradox

Behind the difficulty of integrating data, there is an important yet rarely acknowledged paradox underpinning the logic of mixed methods research that demands some attention. On the one hand, mixed methods designs tend to be seen as either necessary or desirable *because* social phenomena are so complex and multidimensional that they are generally better captured using different modes of exploration. On the other hand, *in spite* of the complexity of the social world, it is assumed that the multiple findings can be integrated and be “put together” to make a coherent picture of the social phenomena.

It follows, therefore, that the question of data integration depends largely on what it is we are “meant” to find with our research questions and subsequent analyses. This is an important point on which much of our argument rests. Yet this necessarily invokes realist antagonisms of what we assume to be “out there” and what is or is not knowable about the social world. As Law (2004) suggests, “If all methods are performative they discriminate by trying to enact realities into and out of being” (p. 148). In other words, the production of social science knowledge is part of the processes through which objects and phenomena come to be known and understood. In the same way, methods have not mysteriously been developed in a vacuum devoid of social context and are as much a product of the world which they seek to describe as the empirical accounts to which they give rise.

If we are to follow this perspective, then turning to the nature of the object of study as well as how mixed methods may also be “performing realities” begs for an interrogation of what we are trying to do when we mix methods in the first place. Simply put:

- Why would a social object of study, say, a place, a process or a phenomenon, which is deemed to be so complex that it warrants more than one method to understand it, be expected to produce different findings that are can be integrated to produce a coherent, knowable whole?
- Is it not the case that, given social phenomena are assumed to be open systems, dynamic, multidimensional, and emergent, we might even *expect* mixed methods to reveal different aspects of entangled phenomena?
- Given mixed methods might be used because of their differences, why would we assume that it is necessarily possible to “integrate” the data, given the methods have already assumed and introduced both multiplicity and difference?

## Messy Cuts: Research Jigsaws?

If we begin with the premise that mixed methods research is needed precisely because the social world is so complex, then it follows that we might also expect to have complex data to deal with—data that may or may not be possible to integrate more or less well. There are many reasons why data may not integrate well in mixed methods research. Be that as it may, there remain instances where, with all the will in the world, irrespective of the strategies used, whichever epistemological frameworks are leaned on, or whatever levels of abstraction are explored, findings from both similar and different methods remain, somewhat unsatisfactorily, as different pieces of (different?) jigsaws that have somehow become jumbled up together, and we cannot make them resemble the picture on the box.

Hacking (1983) makes the point that “most experiments don’t work most of the time. To ignore this fact is to forget what experimentation is doing” (p. 230). Here too, we argue that to ignore the fact that sometimes we cannot integrate or make sense of the mixed methods data is to deny what can often happen with mixed methods research. Our understanding of the social object being studied may become messier, more confused rather than less messy, clearer, or more complete. Furthermore, mixed methods research can splinter and smash up the thing we thought we were studying, and may indeed participate in its transformation. In turn, then, if findings from different methods contradict and trouble each other to the point that it becomes nonsensical to integrate them, then “forcing” data integration by going up a level of abstraction without sufficient evidence to do so, could be committing an epistemological fallacy.

This being the case, following Barad (2007), we can imagine research producing “cuts.” Different methods may produce very different cuts, but the same method may well do too. These cuts produce different “matterings”; they make some aspects visible but not others and this process has social effects. Hence, we begin with the premise that observers and phenomena are always entangled; the methodological cut produces them as separate, and in doing so, produces different forms of visibility and invisibility (Barad, 2007). A cut through an orange makes visible a slightly different surface of the orange’s interior, and, in cutting the orange, the pressure of the knife means that the surface of the orange made visible bears the mark of the cut itself. Yet as each cut reveals and entangles itself within the cuts, perhaps our focus should be less on trying to rebuild a picture of the orange using the data from our cuts, and instead pay attention to the work that the cutting does. We might also begin to consider where we draw the boundaries of the social entity that is an orange: at the box, the tree, the synthetic orange-flavored compound? When piecing the orange cuts together, we have the advantage of knowing what we are meant to be assembling, but what if our methods in fact splice through entangled fruit? Might we be unknowingly bringing together pieces of an orange with pieces of a pineapple and reconstructing entirely new mythical social entities mainly because we are trained to “bring findings together” through integration, whatever the consequences? Indeed, what would it mean if we were to call into question the possibility of a predetermined orange or pineapple and consider the processes through which such distinctions are produced?

In other words, the process of doing research, of making cuts, will always be partial and will always bear the traces of the research process undertaken. Moreover, the act of making these cuts contributes to how we understand what it is we think we are researching. Methods produce objects, and different methods can produce objects that look quite different, even when they are purportedly investigating the same social entity. The challenge of integrating data is often about trying to reconcile some very different-looking “cuts.” Mixed methods, and indeed the same methods at different times and in different spaces, produce different objects too, and those objects have material consequences—the cut has implications beyond the research arena. This is what we mean by “mattering”. Barad (2007) makes this clear in her illustration of the world-making of the cut that is enacted in the use of ultrasound fetal imagery—a cut producing a particular form of visibility that has had political implications through its use in the construction of ideas and imaginaries about mothers and unborn children in the general public and in the political arena. She shows how these visibilities then contribute to a politics of the unborn child that grants agency to a fetus and in doing so removes it from the pregnant woman.

Cuts are boundary-drawing processes that, through what they reveal or conceal, come to matter. Like the apparatuses that Barad (2007) describes, we suggest that methods are the “material conditions of possibility and impossibility of mattering; they enact what matters and what is excluded from mattering”… and in doing so, methods are “boundary-making practices” (p. 148). As she sums up, “knowing is not a bounded or closed practice but an ongoing performance of the world” (p. 149). Within a single study, some findings can be “bounded” across all the methods used; other times, though, particular findings seem to be dislocated and thoroughly bounded in (and out) of particular methodological approaches.

If different visibilities are produced through different cuts, why are we surprised when these “matterings” do not always hold together across methods as we imagine they might? Why do we find it such a problem when mixed methods produce cuts that look like they are from completely different jigsaws? Indeed why do we expect the various cuts to produce something which is capable of integration into a coherent whole at all?

## Acknowledging the Mess

For us, to sometimes refuse to integrate, to let data stand as problematic and multiple with regard to the object of research, is not only absolutely acceptable but it is also at times necessary. Indeed, there is an opportunity for mixed methods to innovate social research, precisely through the way this mode of research often produces different “cuts.” It is the different “cuts” that come to matter in mixed methods, rather than whether all cuts look similar or are produced the same way. These cuts, we argue, can be used to access what is messy, fuzzy, and multiple in the social world (Law, 2004), rather than smoothing-over mess and fuzzy by using a palliative practice of data integration in the production of the coherent research object. As we have argued, all research involves making ‘‘cuts’’. These cuts are the interface between method and object that constitutes both. Furthermore, there are political and social consequences to those research cuts.

In arguing for an approach that acknowledges “mess”, we are not merely suggesting that there are “messy” data sets or “messy” findings. Our argument goes much further than that and follows Law and others (e.g., Byrne, 1998, 2013; Byrne & Callaghan, 2013) in attempting to produce a methodological alternative that explicitly facilitates the empirical engagement with the “complexity” of the social world and thereby underscoring its dynamic, open, nonlinear, emergent, contingent, and multidimensional ontological properties. As Law (2004) puts it:

If the world is complex and messy, then at least some of the time we’re going to have to give up on simplicities. But one thing is sure: if we want to think about the messes of reality at all then we’re going to have to teach ourselves to think, to practice, to relate, and to know in new ways. (p. 2)

It is a tricky predicament. If we start with a basic conceptualization of the object of study and end with the same one, we might argue that we have used a methodological design that is not sufficiently interesting to warrant the research in the first place. Conversely, if we produce findings that completely disrupt or disturb our initial conceptualizations of the object, then we may face the problem of not knowing why the data are as they are, since the data could be revealing something fundamentally new to the researchers or the methods could be “making” particular realities (Law, 2004), which may not be able to be adequately validated. So, we need an alternative “middle ground” where both mess and clarity are made possible through a recognition of the empirical visibilities that come to matter; the diffractive approach we are proposing responds to this.

Mixed methods designs not only offer that opportunity but may be especially interesting when the different data do not integrate well. This is precisely because we see the problems of data integration can thus provide empirical access to the “shifting” and “dislocating” complex, messy and dynamic ontology of the case. An example of where diffraction rather than integration might have been useful is the one that Mol outlines in *The Body Multiple* (Mol, 2002). There, she argues for the concept of “multiple ontologies” due to the range of empirical findings she describes from her detailed ethnographic work. One example she draws on is that of diabetes, which presents itself as an illness with a long list of symptoms, but where individual patients have their own unique configuration of symptoms. Her point is that with an object of study such as “diabetes,” there are multiple ways of “being diabetic”, even if epistemologically there is still “one” list of symptoms or “one” (and now more than one) illness called diabetes.

We can think of multiple instances where particular “outcomes” or “concepts” are presented through multiple and different pathways, where both causality is complex and plural (see Byrne & Uprichard, 2012; Cartwright, 1999, 2004). In such cases, the “thing” being studied is ontologically and epistemologically demanding of a methodological approach that allows for its multiplicity and partiality to be empirically captured in a way that “data integration” does not. This is not about transgressing the norms of mixed methods research or about mess being “better” or more “valid” than nonmess. It is more a question about what happens when “best practice” is followed and yet the data transgresses the researcher(s), the research questions and turns the object of study on its head again.

The problem is, it is impossible to know before gathering data what the findings will be or how easy or hard it will be to integrate different data together. The implicit assumption made in mixed methods research as it stands, though, is that, so long as findings from two or more methods are adequately and/or sufficiently “brought together”, we can “dig deeper” into a “truer truth” of the object. This, by implication, implies that the alternative is somehow “wrong” or “problematic”: that it is the fault of inappropriate choice of methods, poor quality analysis, or problematic data generation; that *if only* we were to use different strategies, then data integration would be possible.

However, there are also times where the problems of integration may lie in part in the *ontological* nuances of what is being studied, and we need a way within social research in general and mixed methods specifically of acknowledging this possibility. Indeed, part of the reason why mixed data are sometimes difficult to integrate is precisely because combining different methods allows us to empirically tap into the dynamic ontologies of social phenomena. After all, all empirical descriptions are made “crisp” or “fuzzy” through methods of making visible: They allow us to expose or conceal the fractured, fragmented, jumbled object of research. It is with this in mind that we argue for a *diffractive* approach to making visible—an approach that acknowledges the reality-making process of the methodological cut, and moreover provides a means for acknowledging the differentiated, complex, and perhaps contradictory facets of the object of research.

## Diffraction

“Diffraction” in physics is the change in direction of waves as they encounter an obstruction or overlap with other waves. Water waves, for example, travel around corners, around obstacles, and through openings, as can be seen in the changing patterns of waves as they come up against boats and walls in a harbor. As Barad (2007, p. 28) puts it, “diffraction has to do with the way waves combine when they overlap and the apparent bending and spreading out of waves when they encounter an obstruction.” Donna Haraway uses the term as an optical metaphor and approach to research and scholarly enquiry that stands counter to “reflection.” As such, its aim is not to represent the object in a different form elsewhere, to make a “new picture” of the research object, but instead to pay attention to patterns of difference, movement, and entanglement.

Diffraction apparatuses study the effects of such interference and difference. In the physics classroom, these apparatuses often shine a laser through slits onto a screen to make visible the diffraction patterns that the light waves and slit produce. They reveal objects in relation and in process, making visible how the process alters the object, rather than reflecting it back to itself. “Diffraction patterns record the history of interaction, interference, reinforcement, difference. Diffraction is about heterogeneous history, not about originals” (Haraway & Randolph, 1997, p. 273).

Barad (2007) further elaborates the concept of diffraction as a scholarly approach, arguing for a diffractive method that makes visible the entanglements of scientific practices and the social. To translate this to social science methodology, diffraction does not assume that there is an unproblematic object that we can simply represent. Diffraction provides a useful alternative to integration; whereas integration assumes that mixed data can be somehow brought together to shed light on a presupposed phenomenon, diffraction emphasizes difference and entanglement. According to Barad (2007),

diffractive methodology is respectful of the entanglement of ideas and other materials in ways that reflexive methodologies are not. In particular, what is needed is a method attuned to the entanglement of the apparatuses of production, one that enables genealogical analyses of how boundaries are produced rather than presuming sets of well-worn binaries in advance. (p. 30)

In other words, the (mixed) data, the object and the (mixed) methods coproduce one another; the ontology of the data, the object, and methodological approach become as important as their epistemologies.

Hence, diffraction is a practice of attending to relationality, process, and messiness in the always-incomplete object, and thinking about how researchers and research practices participate in this becoming-object involves attending to and experimenting with interference patterns, embracing, and indeed playing with contamination and entanglement to see what happens, and to expose the complexity of the world. Diffraction apparatuses are used to produce knowledge about both the object being passed through the apparatus and the apparatus itself. Central to the development of quantum physics, diffraction apparatuses “measure the effects of difference, [and] even more profoundly they highlight, exhibit, and make evident the entangled structure of the changing and contingent ontology of the world” (Barad, 2007, p. 73). In this way, diffraction can be understood as a research practice that acknowledges its participation in world-making, which makes visible its own interference and its various material effects.

We argue that the notion of diffraction, in addition to integration, has much to offer mixed methods research. A diffractive perspective precludes the option of using mixed data to illustrate, enrich or verify another, since that would entail the “holding still” of one and a refusal to see the research object as a messy, processual entity. Diffraction calls into question the very project of integration, instead welcoming the emergence of disjunctures, lacunae, difference, and diversion as a means of troubling the research case as a bounded, isolated unit and revealing the ways in which processes of objectification, the making of the research object, take place.

This is not to say that integration cannot do these things also. However, a diffractive approach assumes ex ante that difference and entanglement recreate the object(s) of study rather than ex post, which integrations tends do. Fetters and Freshwater (2015, p. 115) argue that the “integrative challenge” responds to the “imperative to produce a whole through integration that is greater than the sum of the individual qualitative and quantitative parts.” Conversely, the “diffractive challenge” responds to the imperative to acknowledge that social phenomena can only be partially empirically captured. Diffraction assumes that the whole is always part of something else and that, sometimes, research thoroughly confuses and messes up what we see as the parts and wholes of what we are studying. The distinction is subtle but important.

Diffraction unsettles and producing disturbances. It delves into forgotten material histories, making them partially visible and asking what caused them to forget; it exposes the world in its complexity and messiness. A diffractive approach, then, is about letting the messiness and complexity of the world speak. As Law (2004) puts it “simple clear descriptions don’t work if what they are describing is not itself very coherent. The very attempt to be clear simply increases the mess” (p. 2).

The advantage to diffraction is that it

does not take the boundaries of any of the objects and subjects of these studies for granted but rather investigates the material-discursive boundary making practices that produce “objects” and “subjects” and other difference out of, and in terms of, a changing relationality. (Barad, 2007, p. 93)

Therefore, a diffractive approach in mixed methods research is about thinking with disjuncture; thinking about where data rub up against data and what that can expose about the subject- and object-making that is an inevitable part of the construction of the subject-object-in-the-making. It is about research as participating, experimenting, and inserting ourselves into the world: acknowledging how we may be the diffraction apparatus, the object and the screen all at once (Dawney, 2013, in press). Diffracting is thus a provocation to a status quo that assumes stability of object and/or researcher, revealing the complexity, messiness, and instability of the object.

Importantly, allowing for cuts, mess, and multiplicity does mean that “anything goes.” As Law (2004) puts it:

the absence of singularity does not imply that we live in a world composed of an indefinite number of different and disconnected bodies, atheroscleroses, hospital departments, or political decisions. It does not imply that reality is fragmented. Instead it implies something much more complex. It implies that the different realities overlap and interfere with one another. Their relations, partially coordinated, are complex and messy. (p. 61)

Using a diffractive approach to empirical data cuts enables us to consider the object of research as something which is not necessarily clearly demarcated and bounded, and which may not look like the thing that we imagined it to look like in the first place. Social phenomena have complex histories incorporating material relations, common sense assumptions, existing research norms and practices, ideas about temporality and spatiality, subjects and objects, and political positionings. They are understood differently by differently positioned subjects, through different lenses and in this sense are always multiple. Whereas integration assumes a prior object, diffraction does not; nor does diffraction demand that the object of study remains the same throughout the course of the research. On the contrary, a diffractive approach readily acknowledges the fact that the object of study may become even less bounded or less clear through the empirical process. Given that mixed methods *often* reveal different aspects of the object of study, the question becomes not about how to integrate the data in order to make sense of “the” object of study, but rather: what evidence is there to suggest that the object of study is not complex, multiple, and messy?

## Diffraction Still Needs Integration

This article has interrogated the way that data “integration” is assumed to be the “ideal” outcome for mixed methods research in many (if not all) cases. Given the nature of this discussion, it is worth being absolutely clear: We do not dispute that data from different methods can and should be integrated and interpreted together as much as possible. Rather, we have wanted to suggest that epistemological or methodological issues may not be the only things that prevent this from being possible; the ontological nature of the social itself may also be at the root of the difficulties as well. Data integration may get us some way, but sometimes, a diffractive approach may be necessary instead. Furthermore, as recent more “expansive” approaches have suggested, we have an opportunity in mixed methods research to move away from the assumption that findings need to always and necessarily “cohere” in order to be both valid and reliable. Given the range of mixed data, the sheer multiplicity, volume, and variety of data now available, and advances in philosophy and social theory focusing on the role of practice in making ontologies and epistemologies, there is good reason to consider a slightly different path.

That said, we realize that arguing for a perspective that allows for “mess” and “multiplicity” may be seen as an opportunity by some to refute the importance of data integration. That has not been our purpose – far from it. As noted at the start of this article, the importance of the advances in mixed methods research cannot be underestimated. We do not want to undo the work scholars over the past two to three decades have achieved in transforming particular research communities to finally see mixed methods research as worthwhile and able to produce findings where both quantitative and qualitative methods are given equal weighting. In some fields such as health and education, for example, mixed methods approaches have come on in leaps and bounds.

Nothing we have written here has meant to suggest a return to a singular methodological approach. Rather, what we have wanted to convey is that it is precisely where social objects of study are quintessentially dynamic, complex, and changing, that sometimes findings do not always easily “add up” or integrate easily—and nor should they necessarily expected to. As such, this article offers an opening up of a discussion which questions the assumption that the research “cuts” produced by the different methods *can* and indeed *should* be integrated into a single set of “findings,” or should attempt to faithfully reproduce an object. The issue has not been about using mixed methods; it has been about how we *interpret* the mixed data and the assumption that integration is always and necessarily appropriate.

Social phenomena are necessarily dynamic, nonlinear, multiple and multidimensional, complex, and messy—and studying such social objects may lead to empirical data that do *not* always integrate well. The notion of data integration, as it currently stands in mixed methods research, tends to negate the possibility of any social entity existing as multiple tangled ontologies. Yet, if we are serious about using mixed methods at all, surely it is to use them to disrupt or perturb the status quo and inject a means to capture the rapidly changing, complex practices shaping everyday life.

## Conclusion

There is considerable scope to innovate mixed methods work so that it allows a way into mess, multiplicity, and partial visibilities. A diffractive approach to mixed methods research provides a way of understanding the complexity that emerges from our empirical descriptions, acknowledging times (and places) where mixed data do not “fit” together, and indeed a way of conceptualizing things as not always and necessarily cohering. Allowing for mixed methods research to produce different kinds of empirical snapshots or jagged or blurry-boundaried cuts of an object of study and then exploring how they may be used to expose various different, nonaligning layers may be another way of exploring times (and places) where interventions may be more or less successful.

With this in mind, we have argued for a diffractive approach to analyzing mixed methods data which involves reading the data across methods while allowing data to noncohere, disintegrate and not reproduce objects of study. Consequently, if the data do not hang together and integrate meaningfully, diffraction offers the opportunity to question the case or the methods and indeed the entanglement of the processes of remaking social cases methodologically. Diffraction, then, invites a particular ethos toward mixed methods research—a sort of “attitude.” It is an ethos that does not dismiss the goal of data integration, but rather one that provides new ways to deal with data that do not integrate successfully and that produce incommensurate, confusing, or knotted messy cuts.

Diffraction, we argue, is a way of letting data speak to us in different ways and, conversely, allowing us to speak back with and to the data differently too. Thus, we might extend Fielding’s (2012) point about mixed methods allowing methods to “come into dialogue”, to situation where the dialogue is with new modes of speaking about empirical modes of production in general. Data diffraction, we contend, not only offers an innovative approach to using mixed methods: it also offers a way of responding to research questions with multiple and/or incomplete answers. In doing so, it reflects the ongoing nature of any research problem—its participation in a process that has no clear endpoint—whichever methods are used.

Diffraction allows the data to cohere, or not. And if not, then maybe all there will be to show at the end of the period of research is a number of cuts, producing different visibilities that cannot be forced into a singular narrative. This should not necessarily be seen as a problem. Indeed, these different visibilities each have different political consequences (May, 2005) and leaving the cuts as cuts may enable us to see more clearly those political consequences, especially when viewed alongside other, alternative, cuts. As Barad (2007, p. 179) notes, “cuts cut ‘things’ together and apart. Cuts are not enacted from the outside, nor are they ever enacted once and for all.” Likewise, as an ongoing process toward understanding, the mixed methods research story will continue, as empirical research jigsaws continue to confuse as well as to clarify what it is we are remaking by both integrating and diffracting what it is we may (or may not) be looking at.

## Notes

1. Throughout the discussion that follows, we opt for the term “mixed methods” to emphasize that the argument proposed here refers to research designs that bring together quantitative and qualitative approaches. However, strictly speaking, our argument applies to any study that employs more than one method.
